# QbD product development: rapid optimization and scale-up of PBAE-based siRNA delivery *via* DoE-guided microfluidics

**DOI:** 10.1039/d5pm00379b

**Published:** 2026-01-30

**Authors:** Adrian P. E. Kromer, Laetitia J. M. Eller, David C. Jürgens, Olivia M. Merkel

**Affiliations:** a Department of Pharmacy, Ludwig-Maximilians-Universität Munich Butenandtstrasse 5-13 Haus B 81377 Munich Germany; b Center for NanoScience (CeNS), Ludwig-Maximilians-Universität Munich 80799 Munich Germany; c Ludwig-Maximilians-Universität Munich, Member of the German Center for Lung Research (DZL) Butenandtstraße 5 81377 Munich Germany Olivia.merkel@lmu.de +49 89 2180 77022

## Abstract

Poly(β-amino ester) (PBAE)-based nanoparticles have emerged as promising carriers for RNA delivery, yet clear design rules linking formulation parameters to performance are still lacking. In this study, a Quality by Design (QbD)-guided and Design of Experiments (DoE)-driven approach was combined with high-throughput microfluidics to rapidly identify formulations with favorable physicochemical properties and consistent critical quality attributes (CQAs). Response Surface Modeling revealed that high total flow rates (TFR ≥ 10), nitrogen to phosphorus (N/P) ratios ≥10, and a Flow Rate Ratio (FRR) of 1 : 3 (buffer : ethanol) led to the formation of smaller, more stable particles. Among the polymers tested, a polymer candidate with a balanced composition of hydrophobic and hydrophilic side chains demonstrated optimal intraparticle stability and gene silencing performance. Notably, transfection efficiency depended strongly on formulation parameters beyond polymer type and N/P ratio, with flow rate ratio emerging as a key driver of gene knockdown kinetics. The lead formulation achieved ∼95% gene knockdown even after two weeks of storage at 4 °C. Scale-up production of the lead candidate confirmed the transferability of optimized Critical Process Parameters (CPPs) and preserved CQA profiles, validating the robustness of the design space. This study establishes a robust and scalable QbD-guided workflow for the development of microfluidically manufactured siRNA nanoparticles, enabling rapid optimization, reliable scale-up, and clinically relevant performance.

## Abstract 2

A DoE-screening on an automated microfluidic small-scale device enabled rapid identification of stable PBAE/RNA nanoparticles. Successful transfer of optimized process parameters to a large-scale device confirmed robust and scalable manufacturing.

## Graphical abstract

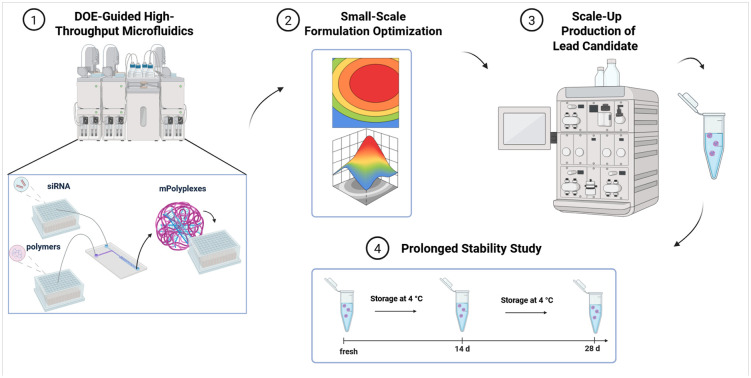

## Introduction

Incorporating Quality by Design (QbD) as early as possible into a product development pipeline becomes more relevant for researchers all over the globe since regulatory authorities demand rational decisions for new formulation development and manufacturing routes.^1–3^ The development of microfluidic technology in nanomedicines has significantly expanded the use of delivery systems in clinical applications.^4–8^ While commonly applied in the manufacturing of lipid nanoparticles, microfluidic manufacturing of polymeric nanoparticles has not yet attracted the same interest in the scientific community and major challenges including transferability remain.^9,10^ Only a limited number of previous studies on microfluidic manufacturing of polymeric nanoparticles have examined the impact of CPPs on biological performance, despite the clear need for in-depth analysis when transitioning from bench to clinic.^11,12^ Here, a well-established poly(β-aminoester) (PBAE) system for siRNA delivery was applied as a case study.^13,14^ This paper presents a framework that serves as a proof-of-concept for transitioning from lab-scale development to GMP manufacturing, reflecting the biopharmaceutical industry's growing reliance on microfluidic systems to streamline both early-stage research and production for product development. Therefore, QbD methodologies were incorporated into the formulation optimization and scale-up of the PBAE platform.

High-throughput microfluidic mixing devices, such as the Sunscreen device (Unchained Labs), allow multiple formulations to be tested simultaneously and significantly reduce processing time.^15^The Sunscreen device works with reusable microfluidic chips, which are designed for durability and can be used from initial screening to process optimization. Reusability reduces consumable costs and ensures consistency throughout the development process.^16^ Notably, this high-throughput device supports nanoparticle manufacturing at high flow rates. It is furthermore optimized for small-scale nanoparticle production. This enables work with small volumes while minimizing material waste, allowing for efficient screening of different formulation parameters, *e.g.* different polymers, N/P ratios, flow rate ratios, as well as total flow rate.^17^

In this context, high-throughput microfluidic devices further enhance time efficiency by enabling rapid screening of multiple formulations.^5,15^ By integrating such advanced technologies with Design of Experiments (DoE), optimal formulations can be identified while minimizing resource consumption and experimental time.^18^

Instead of changing one factor at a time (OFAT), Design of Experiment (DoE) systematically varies multiple factors simultaneously to identify the optimal conditions.^19,20^ This approach reveals interdependencies and combined effects on nanoparticle properties and performance, enabling researchers to predict outcomes across variable combinations.^21^ Additionally, it can reduce the number of necessary experiments drastically, reducing the economic and ecological burden of development processes, while generating deeper understanding and control of processes. This advantage is implemented by identifying the influences of each Critical Process Parameter (CPP) on predefined Critical Quality Attributes (CQA)^19^ and allows a design space with optimal formulation conditions to be defined for an effective PBAE delivery system. These aspects make DoE-based formulation design a highly efficient tool for product development, particularly in fields requiring high reproducibility and quality, such as nanomedicine and pharmaceutical development. Accordingly, DoE is emerging as a key tool for product development in this field.^18,22^

Once optimal formulations are established in preclinical development, the focus shifts to scale-up and to GMP-compliant manufacturing.^19,23,24^ Scaling-up the manufacturing process introduces higher costs and reduces flexibility in process settings. This highlights the necessity to optimize the CPPs as much as possible before starting this critical step.^4^

In this study, optimal formulations, which produce stable particles with favorable CQA profiles, were identified by applying DoE on an automated microfluidic mixing device. Transferring the CPPs to a Knauer NanoScaler, a formulation design transfer to a large-scale production device was demonstrated, effectively confirming the scale-up of the PBAE formulation, which is one of the biggest challenges in formulation development.^25,26^ The CQA profile of scaled-up formulations was retained throughout the process highlighting the robustness of the design space.^27^ This workflow demonstrates a fast and robust optimization and upscaling approach following QbD methodologies, which can be incorporated for any microfluidically manufactured pharmaceutical formulation.

## Materials and methods

### Materials

HEPES (4-(2-hydroxyethyl)-1-piperazineethanesulfonic acid), ethyl trifluoroacetate, sodium chloride, Tris-EDTA buffer solution 100×, RPMI 1640 medium, Triton X-100, heparin sodium salt from porcine intestinal mucosa, heat-inactivated fetal bovine serum (FBS), penicillin/streptomycin solution (P/S), and Dulbecco's phosphate-buffered saline (PBS) were obtained from Sigma-Aldrich (Darmstadt, Germany). Di-*tert*-butyl decarbonate, oleylamine, spermine, dimethylformamide (99.5% pure), Lipofectamine 2000, OPTI-MEM serum reduced medium, 0.05% trypsin-EDTA, Silencer Firefly Luciferase siRNA and non-coding siRNA, and SYBR Gold Nucleic Acid Gel Stain 10000X concentrate in DMSO were purchased from Thermo Fisher Scientific (Schwerte, Germany). 1,4-Butanendiol diacrylate was obtained from TCI Chemical Industry Co., Ltd (Tokyo, Japan). Trifluoroacetic acid (99.9%, extra pure) was purchased from Acros Organics (Geel, Belgium). Methanol-d6 was obtained from Deutero (Kastellaun, Germany). Dichloromethane, methanol, ammonia, potassium permanganate, magnesium sulfate, acetone, pentane, and formic acid (>99% pure) were purchased from VWR Chemicals (Ismaning, Germany).

### Synthesis

PBAE copolymers were synthesized using a well-established method previously reported by our group (settings 1, 16 and 22).^14^ In short, the reaction involved a diacrylate monomer serving as the polymer backbone and two primary amine-containing compounds forming the side chains in varying ratios. Specifically, 1,4-butanediol diacrylate was used as the backbone, while tri-Boc-spermine (TBS) and oleylamine (OA) were incorporated in different proportions. All reactants were dissolved in DMF at a concentration of 300 mg mL^−1^. Following the reaction, the polymers were deprotected with trifluoroacetic acid and subsequently precipitated three times in pentane before final drying. The monomer ratios (hereafter expressed as the percentage of OA in the final polymer) were determined by ^1^H-NMR spectroscopy.

### Preparation of formulations

#### High-throughput microfluidics

Polymers and siRNA solutions were automatically mixed using the Sunscreen high-throughput microfluidics device (Unchained Labs, Pleasanton, CA, USA). The polymer stock solution (1 mg mL^−1^) was prepared in ethanol, while the siRNA stock solution (100 µM) was diluted in HEPES buffer (pH 5.4). All formulations were prepared using a 190X Sunny microfluidic chip (Unchained Labs, Pleasanton, CA, USA), which features an X-shaped cross geometry with a multi-inlet junction and a uniform channel depth of 190 µm, designed to induce laminar-chaotic mixing. Polymer and siRNA solutions were pipetted into two separate 96-well input plates (MASTERBLOCK®, 1 mL, U-bottom; Greiner Bio-One GmbH, Frickenhausen, DE), and the resulting siRNA-loaded PBAE nanoparticles were collected in a 96-well collection plate (Greiner Bio-One GmbH, Frickenhausen, DE) with a collection volume of 800 µL per well. Following the preparation process, the collection well plate was incubated at 80 mbar and 20 °C to remove residual ethanol. After complete evaporation of ethanol, each formulation was reconstituted in HEPES buffer and incubated overnight at room temperature.

### Design of experiment

A reduced combinatorial design was used with the following four factors. Polymer type was used as a categorical factor describing one of three PBAE polymers. Polymers differed in the percentage of hydrophobic side chain ratio from 41% (**H**ydrophilic), over 68% (**B**alanced), to 93% (**L**ipophilic). The N/P ratio was used as a continuous variable with three levels of **7**, **10**, and **13** describing the ratio of positively charged nitrogen atoms in the polymer to the negatively charged phosphates in the siRNA backbone. The total flow rate (TFR) was used as a continuous variable with three levels of 1 (**s**low), 5 (**m**edium), and 10 (**f**ast) mL min^−1^, depicting the combined total flow rate during mixing. The flow rate ratio (FRR) was used as a continuous variable with three levels of 0.25 (**A**queous), 0.5 (**E**ven), and 0.75 (**O**rganic), depicting the proportion of ethanol in the entire flow setup.

The design consisted of 28 experimental runs with duplicates for 4 randomly selected formulations. The duplicated formulations were H7fA, B13fO, L13sO and L7fO. The design was generated using the MODDE 13® (Sartorius, Göttingen, Germany) software package.

Models were generated for the CQAs of nanoparticle characteristics as well as transfection performances and kinetics.

### Scale-up of nanoparticle formulation

For large-scale production of lead formulations of nanoparticles, an impingement jet mixing NanoScaler (Knauer GmbH, Berlin, Germany) was used either at a total flow rate of 10 or 1 mL min^−1^ and a flow rate ratio of 1 : 1 (aqueous to organic) or 3 : 1. Resulting formulations were aliquoted, and ethanol was evaporated at 80 mbar and 20 °C. Volumes were adjusted and formulations were either used immediately or stored at 4 °C.

### Nanoparticle characterization

The hydrodynamic diameter (DH) and polydispersity index (PDI) of the obtained nanoparticles were determined by dynamic light scattering (DLS) using a Zetasizer Advance Ultra (Malvern Instruments, UK) with 3 measurements per sample at a backscatter angle of 173°.

### Determination of intraparticular stability

The stability of the resulting nanoparticles was evaluated by using a modified polyanion competition assay. Briefly, mixtures of Triton-X and heparin at varying concentrations were applied to induce siRNA release from the nanoparticles. In a black 384-well plate, 10 μL of nanoparticle suspension was mixed with 20 μL of the corresponding Triton-X–heparin stress solution. Each sample was measured in triplicate. Eight different stress-solution concentrations plus a blank were used per nanoparticle suspension. After the stress solutions were added, plates were sealed to avoid evaporation and incubated at 37 °C at 150 rpm for 1 h. Afterward, 5 μL of a 4× SYBR Gold dye was added to the mixture and incubated for 5 min in the dark. Finally, the fluorescence was measured using a TECAN Spark plate reader (TECAN, Männedorf, Switzerland) at 492 nm excitation and 537 nm emission wavelengths. Using GraphPad Prism 10.5.0 software, a nonlinear fit/logarithmic fit was performed to calculate the EC50 values of each polymer relative to the maximum released siRNA in each sample.

### Cell culture

H1299-luciferase cells were prepared and cultured as described elsewhere.^28^ They were maintained in RPMI-1650 medium (Sigma Aldrich, Taufkirchen. Germany) supplemented with 10% (v/v) fetal bovine serum (FBS) (Thermo Fisher Scientific, Darmstadt, Germany). Cells were incubated at 37 °C in a sterile environment with 95% relative humidity and 5% CO_2_.

### Cell transfection

H1299-luciferase cells were seeded in 48-well plates at a density of 2500 cells per well in 100 µL of culture medium and incubated for 24 h or 48 h at 37 °C under 5% CO_2_. For transfection, 20 µL of each suspension of Sunscreen-formulated siRNA-loaded PBAE nanoparticles (corresponding to 100 pmol siRNA per well) was added directly to the cells in each well. Each sample was measured in triplicate. For the NanoScaler-formulated nanoparticles, a dose-response transfection was performed using 5 nM to 50 nM siRNA per well.

### Cell lysis

At 24 or 48 hours post-transfection, the medium was aspirated, and cells were washed once with 200 µL of PBS per well. PBS was removed, and 100 µL of 1× cell lysis buffer was added to each well. Plates were incubated at room temperature for 1 hour.

### Firefly luciferase glow assay

After lysis, 35 μL of cell lysate was transferred to each well of a 96-well white plate to measure luciferase activity. After adding 100 μL of LAR buffer (20 mM glycylglycine, 1 mM MgCl_2_, 0.1 mM EDTA, 3.3 mM dithiothreitol (DTT), 0.55 mM adenosine triphosphate (ATP), 0.27 mM coenzyme A; pH 8.5) supplemented with 5% (v/v) of a mixture of 10 mM luciferin and 29 mM glycylglycine to each well, the luminescence counts were measured for 10 seconds using the Tecan plate reader. Luminescence signals were normalized to the total protein content per well as determined using the Pierce BCA Protein Assay Kit (Thermo Fisher Scientific, Rockford, IL, USA). Gene knockdown of each siLUC PBAE formulation was calculated by normalizing the luminescence values with those of cells treated with the respective negative control (siNC) PBAE formulation.

## Results and discussion

### Drafting the design space

To generate reliable DoE models, the reproducibility of a process needs to be high, and user-dependent variance needs to be minimized. This is why the Sunscreen formulation screener was used for this study. The device allows fast and highly reproducible formulation screening. A quadratic reduced combinatorial design drafted with Modde® 13 was utilized to optimize three continuous and one categorical factor (Table 1). To investigate process reproducibility, duplicate runs were incorporated into the design. As a categorical factor, three different types of PBAE polymers with varying hydrophobicity were introduced. PBAE polymers with 41% of hydrophobic side chains (**H**ydrophilic), 68% (**B**alanced), and 93% (**L**ipophilic) were investigated. As continuous factors, the following CPPs were incorporated: (i) the N/P ratio, depicting the number of positively charged nitrogen atoms in the polymer against the negatively charged phosphate groups in the siRNA backbone (**7**, **10**, and **13**); (ii) the total flow rate (TFR) at 1 mL min^−1^ (**s**low), 5 mL min^−1^ (**m**edium), and 10 mL min^−1^ (**f**ast); and finally (iii) the flow rate ratio (FRR) between the organic ethanol phase containing the polymers and the aqueous buffer containing the siRNA of 0.25 (**A**queous), 0.5 (**E**ven), and 0.75 (**O**rganic). The entire design is depicted in Table S1. To facilitate the comparison between runs, formulations are named after their CPP with *e.g.* B7 sE referring to the balanced polymer B at N/P 7 mixed at a TFR of 1 mL min^−1^ and a FRR of 0.5.

**Table 1:** Parameter settings and abbreviations for the reduced combinatorial design space

Parameter	Polymer	N/P ratio	TFR	FRR
Abbreviation	93% = **L**ipophilic	**7**	1 = **s**low	0.25 = **A**queous
	65% = **B**alanced	**10**	5 = **m**edium	0.5 = **E**ven
	41% = **H**ydrophilic	**13**	10 = **f**ast	0.75 = **O**rganic

### Characterizing colloidal and intraparticular stability

**Fig. 1:**
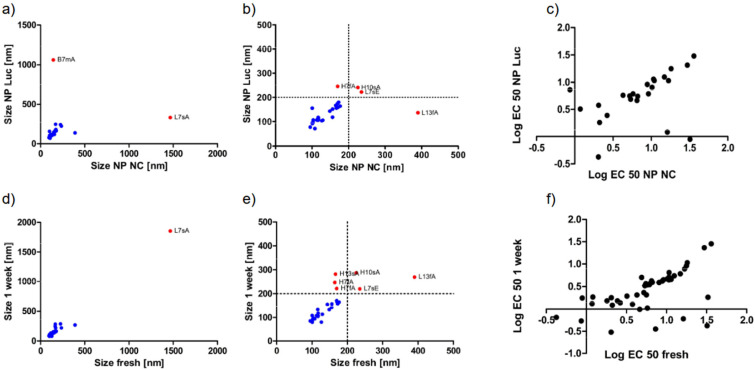
Physicochemical characteristics of nanoparticles prepared with a PBAE library. Hydrodynamic diameter of PBAE formulations prepared with siLUC *vs.* siNC for the entire library (a), and for formulations below 500 nm (b). Log EC 50 stability values of nanoparticles prepared with siLUC *vs.* siNC (c), hydrodynamic diameter of PBAE formulations prepared with siLUC for freshly prepared particles *vs.* 1 week of storage at 4 °C (d), and for formulations below 500 nm (e). Log EC 50 stability values of nanoparticles prepared with siLUC or siNC and stored for 1 week at 4 °C (f).

In the field of siRNA delivery, it is widely accepted that different RNA sequences exert only minor effects on the formulations when they share the same length and modification profiles. Nonetheless, siRNA sequence was included as an additional experimental factor, and all formulations were prepared using either siRNA targeting firefly luciferase (siLUC) or a scrambled, negative-control siRNA (siNC). As shown in Fig. 1, the siRNA sequence played a minor role regarding the size of the nanoparticles, and most formulations exhibited favorable size profiles below 200 nm, with strong correlation between nanoparticles formulated with siNC and siLUC. PDI values showed weaker correlation but were mainly in a favorable range below 0.4 (Fig. S1).

Formulations with poor size profiles such as L7sA also exhibited poor correlation between siNC and siLUC. This could be an indication of particle aggregation, which occurs randomly and heterogeneously in each preparation run, leading to poor reproducibility. Additionally, laser diffraction scanning reproducibility is reduced when measuring polydisperse or aggregated samples due to their slow and heterogeneous diffusion profiles, also affecting reproducibility.

Previous studies reported that intraparticular stability is an important parameter to predict the *in vitro* nanoparticle performance.^29^ To investigate this parameter, a competition assay based on heparin and Triton-X was developed to assign stability values to each nanoparticle formulation. As seen in Fig. 1a–c, stability correlated strongly between particles prepared with siLUC and siNC, indicating minimal impact of the siRNA sequence on the intraparticular stability. In a previous study, it was found that formulations with stability values above 1.5 would not be able to mediate a significant gene knockdown.^29^ It was hypothesized that excessively high intraparticle stability would hinder cargo release in the endosome and therefore fail to mediate cytosolic delivery. Interestingly, all formulations in this design revealed stability values below this threshold.

The storage stability of a formulation is an important quality attribute and is seldomly investigated in the field of polymeric nanoparticles. To investigate the colloidal stability of the formulations, they were stored for one week at 4 °C, and subsequently size and intraparticular stability were measured. As presented in Fig. 1d,e and Fig. S1–S3, size, PDI and stability data of freshly prepared particles correlated strongly with the results obtained after one week of storage. For the size measurements, it can be observed that particles larger than 200 nm deviated from the correlation. This implies that particles with poor initial size profiles also demonstrate poor storage stability. Nonetheless, most formulations were stable over at least one week at 4 °C.

### Modeling colloidal and intraparticular stability

**Fig. 2:**
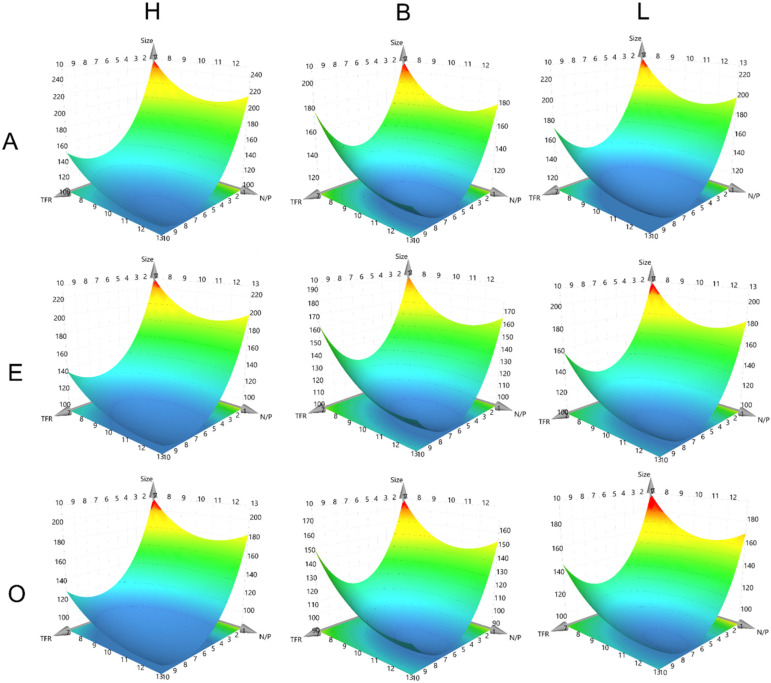
RSM of particle hydrodynamic diameter. *X*-Axes depict N/P ratios, *Y*-axes depict TFR, horizontal alignment depicts polymer type and vertical alignment depicts FRR. *Z*-Axes depict the model response.

**Fig. 3:**
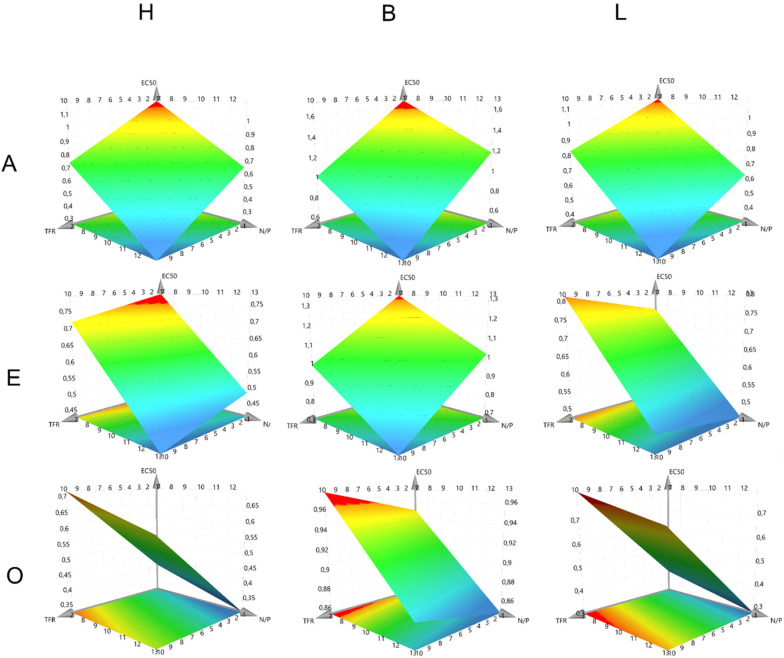
RSM of intraparticular stability. *X*-Axes depict N/P ratios, *Y*-axes depict TFR, horizontal alignment depicts polymer type and vertical alignment depicts FRR. *Z*-Axes present the model response.

A central part of a QbD approach is the development of a design space (DS) in which a process is fully understood and controlled. In DoE methodologies, a common tool to achieve this is the Response Surface Methodology that provides response surface models (RSMs), which establish equations that can predict all fitted CQAs for every possible combination of different CPP settings. Fitting is mostly performed using multiple linear regression (MLR) or partial least squares (PLS). Here, PLS was used to fit the CQAs of fresh particle size, particle size after one week, intraparticle stability, and intraparticle stability after one week (Fig. 2, 3 and Fig. S4, S5). For all CQAs, fitting models were derived using *R*^2^-values above 0.75 for the size models and above 0.65 for the EC50 models (Fig. S6). As expected, the siRNA sequence was not a significant factor in the size model (Fig. S7).

The RSMs reveal that the found trends for TFR and N/P ratio remained similar over all FRR settings and polymer types. This was shown through the similar shape of the RSMs in all plots. The RSMs also reveal that higher TFR and an N/P ratio equal to or above 10 were required to generate the smallest particles. Higher TFR leads to turbulent mixing, which can generate closer proximity between the polymer and siRNA cargo before nanoparticle formation occurs due to the hydrophobic attraction forces within the polymers. Another potential reason for the smaller size when the N/P ratio is higher might be the increased particle number, which is a result of more polymer becoming rapidly available, decreasing the number of siRNA molecules per nanoparticle and with it the average size, while increasing the overall particle number.

It was also demonstrated that the FRR “**O**” setting, meaning a ratio of buffer to ethanol of 1 : 3, led to the smallest particle sizes, which can be appreciated on the vertical comparison of the *Z*-axes of the plots. Interestingly, this trend contradicts the commonly accepted manufacturing routine for LNPs, which corresponds to the “**A**” settings. But it aligns with the rationale for the N/P ratio. If the polymer is distributed in higher volumes, the available polymer in the proximity of a single RNA molecule is reduced, potentially reducing the resulting particle size while simultaneously increasing the particle number. Independent of FRR, **B** polymer generally produced the smallest particles on average, followed by the **L** polymer and finally the **H** polymer. The dominant stabilizing forces within the particles are electrostatic attraction and hydrophobic interactions. The balanced polymer likely represents an optimal compromise between both, resulting in more tightly packed and therefore smaller particles. Interestingly, the more lipophilic polymer produced smaller particles compared with the hydrophilic one, which suggests hydrophobic interactions are more dominant than electrostatic interactions. However, this interpretation is unlikely. In previous publications, we established the so-called micelle-embedded polyplex (mPolyplexes) structure of these particles using TEM imaging and molecular dynamics simulations.^30^ The reported structure showed that siRNA is first encapsulated in an inverse micelle where the hydrophilic parts of the polymer are oriented toward the hydrophilic siRNA core and the hydrophobic parts are oriented toward the outside. Several of these inverse micelles coalesce into a larger particle which becomes the mPolyplex. Coalescence is mainly driven by the hydrophobic proportion of the polymer, which could explain the smaller particle size for the more hydrophobic **L** polymer.

After 1 week of storage, all response surface models showed the same trends as observed for the fresh particles (Fig. S4) with almost unchanged numbers, confirming colloidal stability over the tested time period.

The RSMs for the intraparticular stability confirmed the hypothesis of the **B** polymer representing the optimal compromise between electrostatic and hydrophobic stabilizing forces, with the overall strongest stability values, independent of FRR. These findings align with the previous size findings for the other polymers as well. Surprisingly, lower N/P ratios tend to result in higher stability values, which could again be associated with the general number of siRNA molecules per nanoparticle. A lower N/P ratio results in more siRNA per polymer within the particles and thereby larger nanoparticles. Larger particles can resist the heparin and Triton-X competition better since they have a more favorable surface area to volume ratio. Interestingly, an interaction between the TFR and the FRR was found, reflected by the rotation of the RSMs when increasing the FRR (moving vertically from A to O). The stability values of the nanoparticles decreased when a more aqueous system was mixed at high TFRs and the stability values increase when a more organic system is mixed at high TFRs. This trend was observable for all polymers and could again be explained by the coalescence of the mPolyplexes. As discussed above, coalescence is an important factor influencing particle size and stability. At high TFR, turbulent mixing rapidly brings siRNA and polymers into close proximity, promoting fast formation of inverse micelles. In more aqueous environments, particles are more prone to rapid coalescence than in more organic ones, which may lead to less optimal and thus less stable packaging. This kinetic effect may explain the greater stability of particles prepared in more organic fluids, where slower assembly enables more ordered and stable structures.

### Transfection efficiency

All formulations were evaluated in H1299 Luc cells with respect to gene knockdown (KD) efficacy. Cells were either treated with the formulation containing siNC as a reference or with siLUC. Additionally, the knockdown was evaluated 24 h and 48 h post-transfection to evaluate potential differences in the knockdown kinetics. Duplicates from the DoE Library were evaluated as well to check for experimental reproducibility. Interestingly, a broad spectrum of KD efficiencies was found, which sometimes varied even within the same polymer and N/P group. For example, H13sA mediated less than 20% KD and H13fE achieved more than 90% KD (Fig. S8). This difference was surprising since the common belief is that the main factors governing the efficiency of a polymeric siRNA delivery system are the nature of the polymer itself and its N/P ratio. Formulation-dependent (TFR and FRR) performance differences of this magnitude are not commonly reported in the literature. The second surprising finding was that stark differences were observed between the 24 h and 48 h knockdown values in some formulation groups. For example, the KD of B10mA after 48 h was almost twice as large with over 65% than the 24 h KD with roughly 30%, while B7mO, prepared with the same polymer, achieved less than half the KD efficacy after 48 h compared with the 24 h values (Fig. S9). These differences in time-dependent KD efficiencies were observed for all three polymer types and indicated that the kinetics of gene knockdown not only depended on the polymer type but also on the formulation method (Fig. S8–S10).

**Fig. 4:**
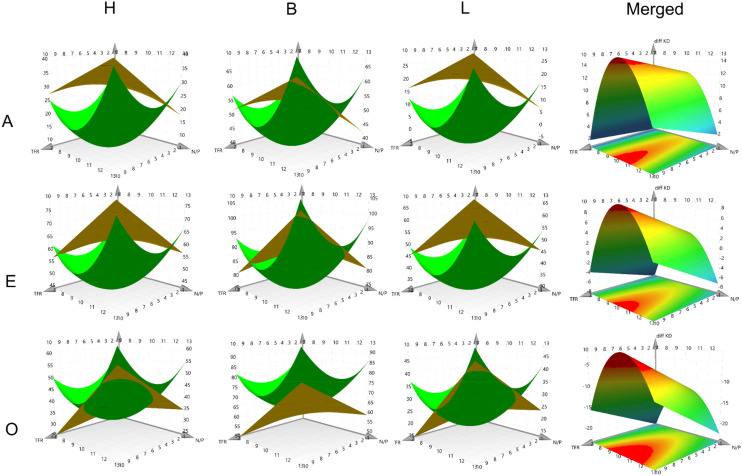
RSM of gene knockdown in H1299 Luc cells. Green surfaces present gene knockdown after 24 hours and yellow surfaces present gene knockdown after 48 h. *X*-Axes depict N/P ratios, *Y*-axes depict TFR, horizontal alignment depicts polymer type and vertical alignment depicts FRR. *Z*-Axes present the model response. Merged graphs depict the model of differences in gene knockdown, which follows the previously described schematic.

To gain more insights into the impact of formulation on KD kinetics and efficacy and to follow the Quality by Design objective, RSMs for the 24 h and 48 h KD values were generated separately (Fig. 4 and Fig. S11, S12). Notably, the response surfaces differed markedly depending on the process parameters, with varying intersections between surfaces. An intersection corresponds to sustained knockdown over the full 48 h period. When the 48 h surface (yellow) lies above the 24 h surface (green), knockdown kinetics are slow, and maximal silencing is reached after 24 h. Conversely, when the 24 h surface exceeds the 48 h surface, knockdown occurs more rapidly, peaks before 48 h, and is followed by recovery of gene expression. For clarity, merged RSMs were generated by subtracting the 24 h KD from the 48 h KD. The resulting surfaces are shown in Fig. 4 (merged column). Positive *Z*-axis values indicate slow KD kinetics, since the 48 h KD was higher. Values near zero reflect stable KD between 24 and 48 h, and negative values indicate fast KD kinetics, since the 24 h value was higher.

The models indicated that the main parameter explaining the differences between slow and fast KD kinetics was the FRR (Fig. S12). A more aqueous mixing system was associated with slower KD while a more organic mixing system was associated with faster KD kinetics, and an even system was best suited for stable KD kinetics between 24 and 48 h. Further investigation is required to elucidate these mechanisms, as overall KD kinetics are influenced by multiple processes, including cellular uptake, endosomal escape, cargo release and target half-life.^31^ These questions were outside the scope of this study but will be investigated further in the future.

These findings demonstrate that a well-defined quality strategy and precise design-space control enables modulation of cellular gene expression kinetics.

### Prolonged stability

**Fig. 5:**
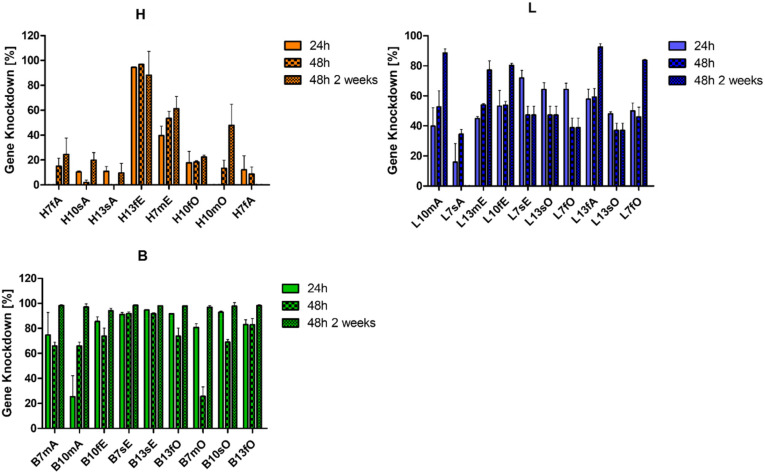
Gene knockdown efficacy of polyplexes prepared with hydrophilic (**H**), lipophilic (**L**) or balanced (**B**) polymers as a function of formulation parameters shown for 24 and 48 hours of incubation and 48 hours of incubation after 2 weeks of storage (error bars depicting standard deviation with technical replicates of *n* = 3).

Motivated by the colloidal and intraparticular stability values, particles were stored for 2 weeks at 4 °C. Hydrodynamic diameters were determined again (Fig. S13) before testing the 48 h transfection efficiency. As shown in Fig. 5, all trends observed for polymer **H** were comparable to trends observed with the freshly prepared particles, indicating that particles were indeed stable for at least 2 weeks of storage. In the case of polymer **L**, formulations L7sE, L13sO, L7fO, and L13sO showed no impact from storage. However, all other formulations of polymer **L** showed increased performance after storage. Most surprisingly, all polymer **B** formulations mediated the same KD of roughly 98% before and after 2 weeks of storage. These overall findings suggest that in polyplexes prepared with some polymers, a rearrangement takes place during the storage time towards an energetically preferred form. Interestingly, this phenomenon does not occur in every formulation of the **L** polymer, which excludes pure hydrophobic rearrangements as driving forces. However, recent work from our laboratory showed that interactions between hydrophobic polymer subunits and the endosomal membrane are critical for endosomal escape.^32^ One possible explanation is that structural rearrangement exposes additional hydrophobic side chains to enhance subsequent interactions with the endosome.

### Scale-up of lead formulations

In line with the QbD framework, a formulation from the most promising and robust design space region (B7sE) was selected for scale-up experiments. To validate the approach, a second formulation based on a different polymer (L13fA) was also evaluated. Following design transfer, both formulations were prepared using the NanoScaler, while maintaining identical polymer type, N/P ratio, TFR and FRR as in the Sunscreen trials. Particle size and intraparticular stability were comparable between NanoScaler and Sunscreen formulations (Fig. S14.).

**Fig. 6:**
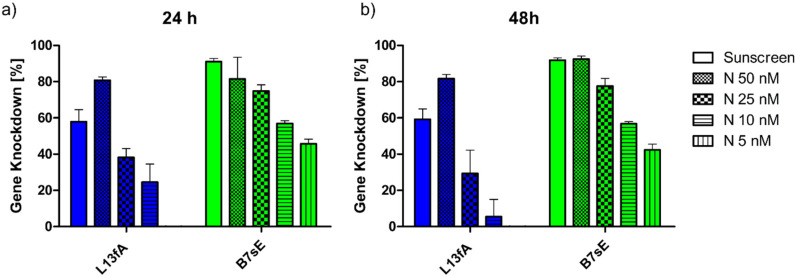
Gene knockdown values for lead formulations prepared with the Sunscreen device (open bars) or the NanoScaler (patterned bars, different concentrations) microfluidic mixing device in H1299 Luc cells after 24 h (a) and 48 h (b) of incubation (error bars depict standard deviation with technical replicates of *n* = 3).

The KD efficiency after 24 h and 48 h was evaluated with the NanoScaler formulations at the same concentration as the Sunscreen formulations (50 nM) and across a dose range down to 5 nM (Fig. 6). No significant differences in KD efficiency were observed between B7sE formulations produced with the Sunscreen device and NanoScaler at either time point, validating the rapid and efficient scale-up, enabled by the QbD-driven workflow.

Notably, the lead formulation achieved about 40% KD even at the very low dose of 5 nM and maintained this effect after both 24 h and 48 h. Formulations stored for 2 or 4 weeks at 4 °C, or for 4 weeks at −20 °C, exhibited the same performance as that for freshly prepared particles, demonstrating their excellent colloidal stability profiles (Fig. S15–S23). These results underscore how QbD and DoE enable rapid development of a stable, clinically scalable lead formulation.

## Conclusion

This study highlights how DoE can be used to define a validated design space that enables the identification of key CPP and optimal formulation conditions for lead candidate product development. Combined with high-throughput microfluidics, this integrated approach enabled a highly efficient screening platform that tested multiple formulations while minimizing material use and experimental time.

Here, higher total flow rates (TFR) and N/P ratios of ≥10 consistently produced smaller particles, while a buffer-to-ethanol FRR of 1 : 3 yielded the smallest particles. Among the polymers tested, the balanced polymer emerged as the most favorable candidate as it combines reduced smaller sizes with the greatest intraparticle stability across all FRR conditions. This strong impact of formulation parameters on transfection efficiency is unprecedented to the best of our knowledge. Pronounced differences between 24 h and 48 h knockdown efficiency further revealed that gene silencing kinetics is governed not only by polymer composition but also by the formulation process. Notably, FRR was identified as the primary determinant distinguishing fast from slow knockdown kinetics.

Importantly, scale-up experiments of the lead formulation (B7sE) yielded no significant differences in knockdown efficiency at 24 h or 48 h between particles produced with the Sunscreen device and the NanoScaler. These results confirm consistency and robustness between high throughput and scale-up production platforms. Moreover, B7 sE achieved >90% gene KD, even at the very low siRNA dose of 50 nM, with effects maintained at both time points (24 h and 48 h).

It should be emphasized that the present work was primarily directed toward product development, with the proposed approach intended to illustrate the practical efficiency of Quality by Design (QbD) principles. While more detailed mechanistic investigations would be feasible, these were beyond the scope of the current study. This is reflected, for example, in the categorical treatment of polymer types, which inherently neglects certain physicochemical properties. Moreover, depending on the intended application, a more comprehensive *in vitro* evaluation employing additional cell lines may be required. For other use cases, an expansion of the design space may also be necessary. In this context, parameters such as alternative microfluidic chip architectures, buffer systems, organic solvents, siRNA modifications, and further formulation- or process-related factors could be systematically assessed using an approach analogous to that presented here. Cytotoxic evaluation and optimization of the reported polymers is a topic of ongoing studies and will be reported elsewhere.^29^

In conclusion, stable nanoparticles with favorable CQA profiles were rapidly generated using high-throughput microfluidic mixing, enabling efficient formulation optimization with minimal material and time effort. Optimized CPPs were successfully transferred to a large-scale platform, confirming both scalability and consistency of CQA profiles. These findings demonstrate a robust, QbD-based strategy for rapid optimization and scale-up of microfluidic pharmaceutical formulations.

## Conflicts of interest

Olivia M. Merkel is a Co-Founder of RNhale GmbH, a Scientific Board Member for Coriolis Pharma GmbH, AMW GmbH, Corden Pharma International GmbH, and an Advisor for PARI Pharma GmbH, Boehringer-Ingelheim International GmbH, and AbbVie Deutschland GmbH on unrelated projects. Adrian P. E. Kromer is an independent consultatnt for AMW GmbH. The remaining authors declare that they have no known competing financial interests or personal relationships that could have appeared to influence the work reported in this paper.

## Supplementary Material

PM-003-D5PM00379B-s001

## Data Availability

The data supporting this article have been included as part of the supplementary information (SI). Supplemantary information contains details on the DoE* *combinatorial design, hydrodynamic diameters, PDI, RSM of hydrodynamic diameters and intraparticular stability, Observed *vs*. Predicted plots, Coefficient plots, and gene knockdown. See DOI: https://doi.org/10.1039/d5pm00379b.
