# Supplementary material for: QbD product development: rapid optimization and scale-up of PBAE-based siRNA delivery via DoE-guided microfluidics
Source: RSC Pharm. 2026 Jan 30;3(2):379–89. doi: 10.1039/d5pm00379b (PMC12926864; doi:10.1039/d5pm00379b)
Supplement: PM-003-D5PM00379B-s001 [file PM-003-D5PM00379B-s001.pdf]

Supplementary Information (SI) for RSC Pharmaceutics.
This journal is © The Royal Society of Chemistry 2026

Supplementary Information

QbD Product Development: Rapid Optimization and Scale-Up of PBAE-Based siRNA Delivery via DOE-Guided Microfluidics

Adrian P.E. Kromer[1*], Laetitia J.M. Eller[1*], David C. Jürgens[1] and Olivia M. Merkel[1,2,3]

Corresponding author:
Prof. Dr. Olivia Merkel
LMU Munich
Butenandtstraße 5
81377 Munich
Germany
0049 89 2180 77022
Olivia.merkel@lmu.de

[1]Department of Pharmacy, Ludwig-Maximilians-Universität Munich, Butenandtstrasse 5-13, Haus B, 81377 Munich, Germany

[2] Center for NanoScience (CeNS), Ludwig-Maximilians-Universität Munich, 80799 Munich, Germany

[3] Ludwig-Maximilians-Universität Munich, Member of the German Center for Lung Research (DZL)

*A.P.E.K. and L.J.M.E. contributed equally to this work.

Keywords

PBAE polymers, siRNA Delivery, Quality by Design, Design of Experiment, Scale Up

Supplementary Table S.1.: Reduced combinatorial design used for the microfluidic formulation optimization with respective nomenclature. Design was carried out with siLUC and with siNC.

NAME	TFR	POLYMER	N/P	FRR
**B7MA**	5	68	7	0.25
**L10MA**	5	93	10	0.25
**H7FA**	10	41	7	0.25
**H10SA**	1	41	10	0.25
**H13SA**	1	41	13	0.25
**L7SA**	1	93	7	0.25
**B10MA**	5	68	10	0.25
**H13FE**	10	41	13	0.5
**H7ME**	5	41	7	0.5
**B10FE**	10	68	10	0.5
**L13ME**	5	93	13	0.5
**B7SE**	1	68	7	0.5
**L10FE**	10	93	10	0.5
**B13SE**	1	68	13	0.5
**L7SE**	1	93	7	0.5
**H10FO**	10	41	10	0.75
**L13SO**	1	93	13	0.75
**L7FO**	10	93	7	0.75
**H10MO**	5	41	10	0.75
**B13FO**	10	68	13	0.75
**B7MO**	5	68	7	0.75
**B10SO**	1	68	10	0.75
**H13MO**	5	41	13	0.75
**L13FA**	10	93	13	0.25
**H7FA**	10	41	7	0.25
**L13SO**	1	93	13	0.75
**L7FO**	10	93	7	0.75
**B13FO**	10	68	13	0.75

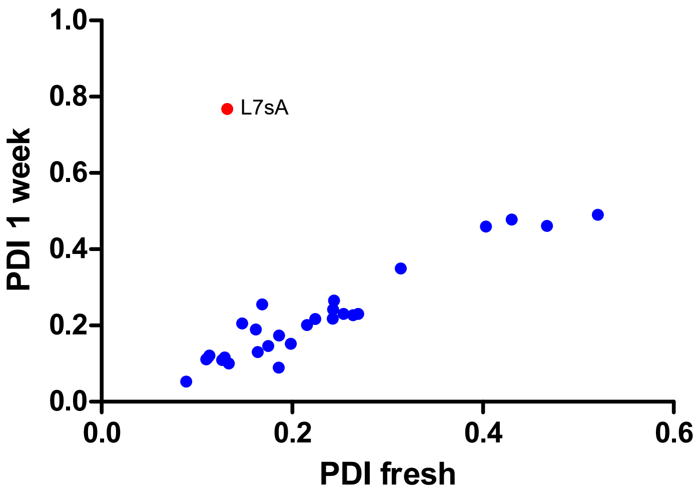

Supplementary Figure 1.: PDI comparison of freshly prepared particles with siLuc and siNC versus particles stored for one week at 4°C.

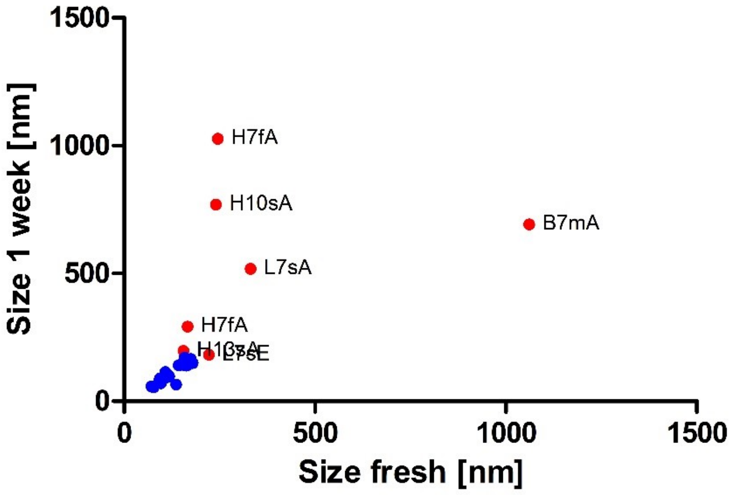

Supplementary Figure 2.: Size comparison of freshly prepared particles with siNC versus particles stored for one week at 4°C.

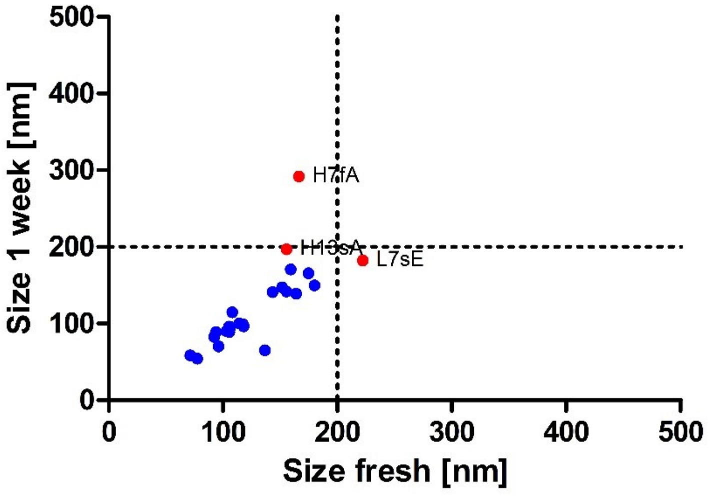

Supplementary Figure 3.: Size comparison of freshly prepared particles with siNC below 500 nm against particles stored for one week at 4°C. Red dots depict particles above 200 nm.

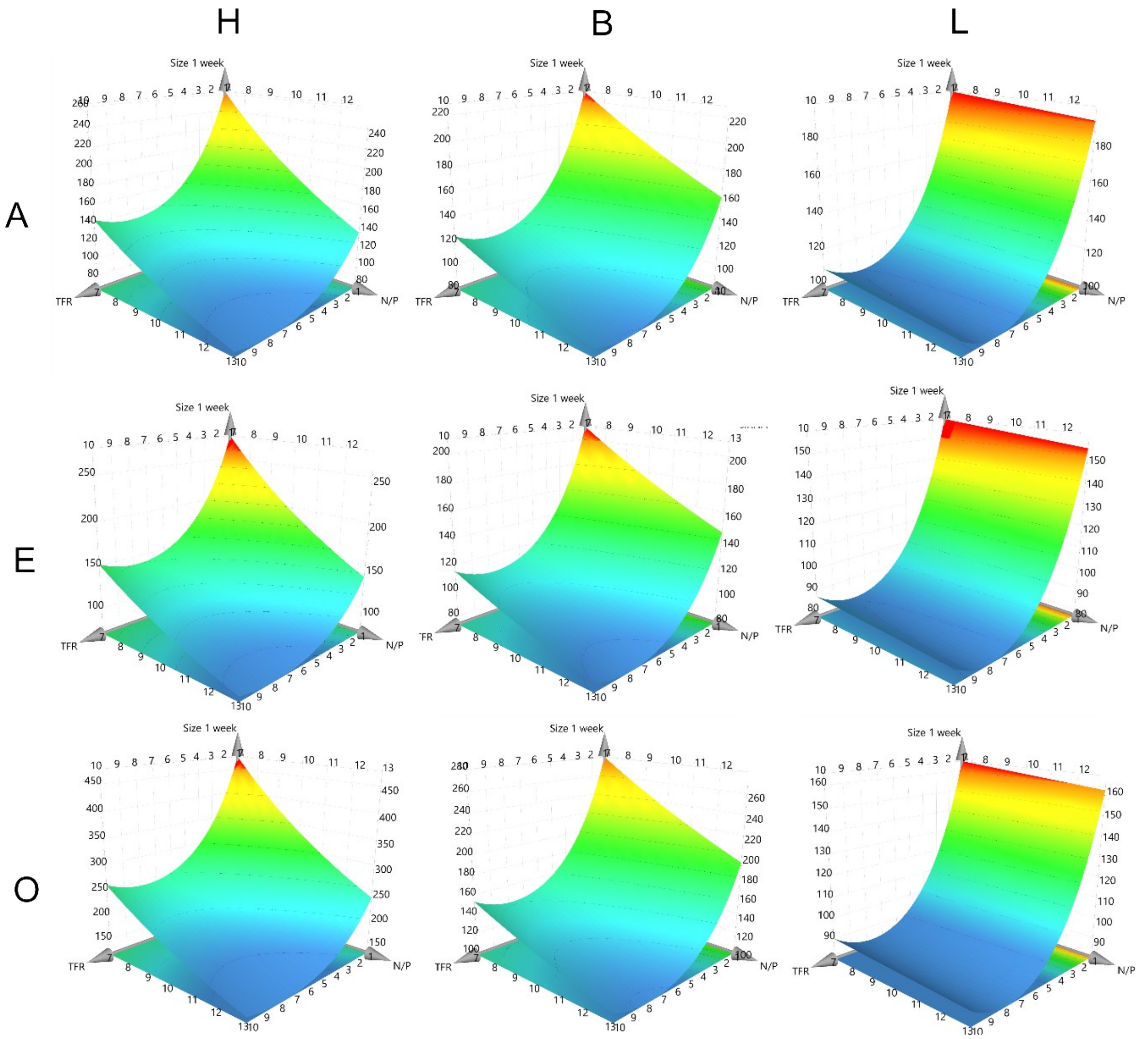

Supplementary Figure 4.: RSM of particle hydrodynamic diameters after 1 week of storage at 4°C. X-axes depict N/P ratios, Y-axes depict TFR, horizontal alignment presents polymer type and vertical alignment presents FRR. Z-axes depict the model response.

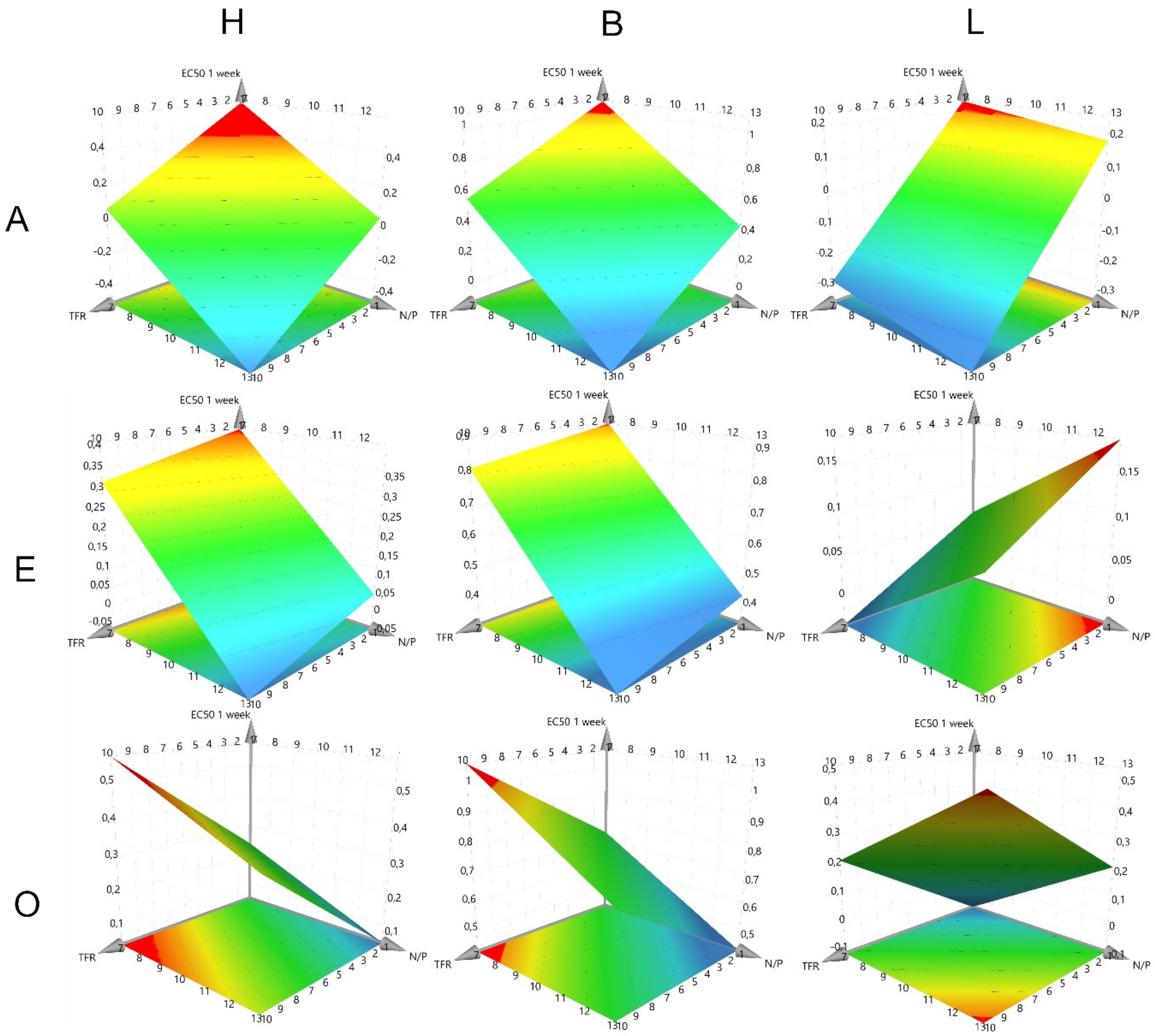

Supplementary Figure 5.: RSM of intraparticular stability after 1 week of storage at 4°C. X-axes depict N/P ratios, Y-axes depict TFR, horizontal alignment presents polymer type and vertical alignment presents FRR. Z-axes depict the model response.

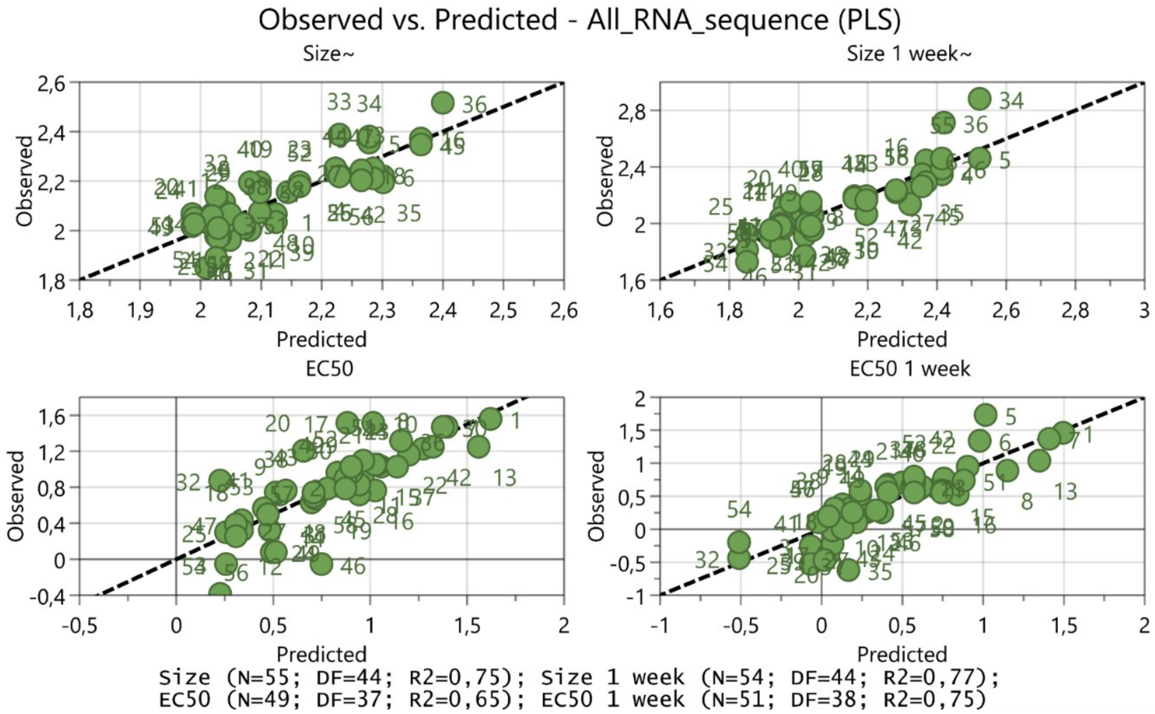

Supplementary Figure 6.: Observed vs. Predicted plots for the Size and EC50 model of freshly prepared particles and particles which were stored for one week at 4°C containing samples with both siRNA sequences.

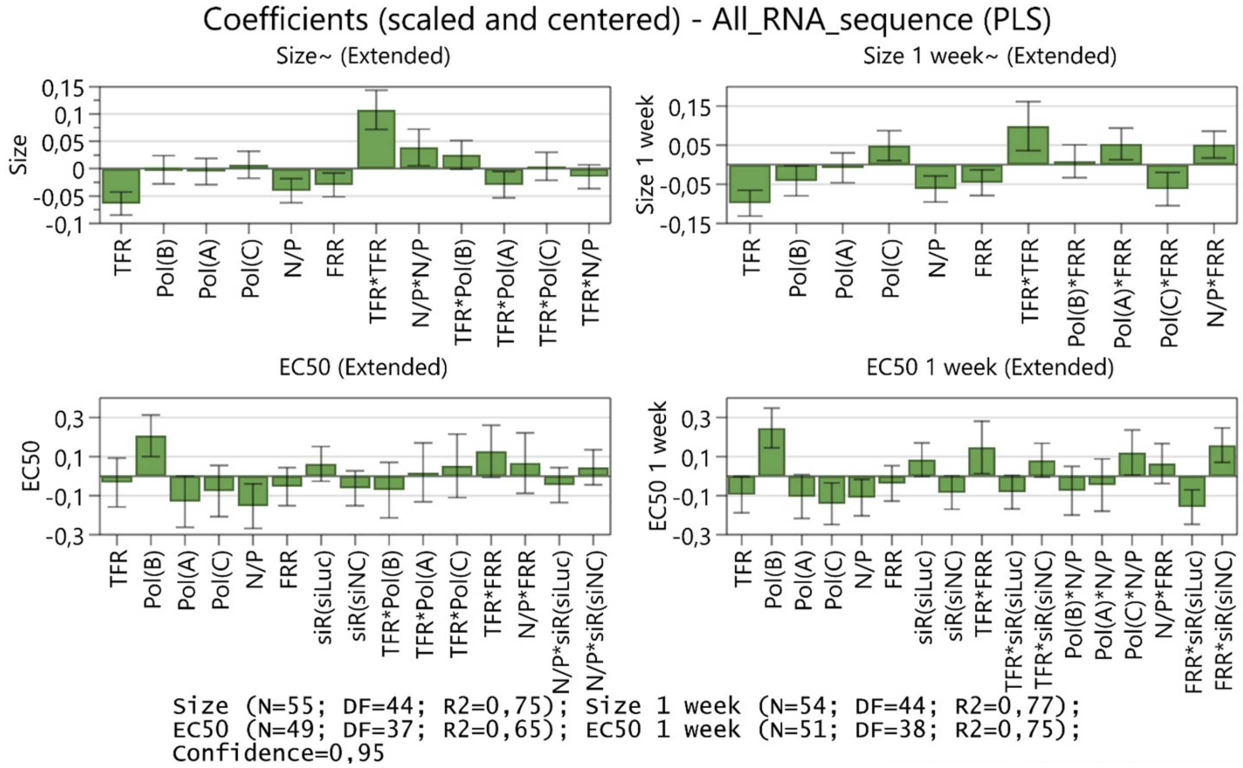

Supplementary Figure 7.: Coefficient plots for the Size and EC50 model of freshly prepared particles and particles which were stored for one week containing samples with both siRNA sequences.

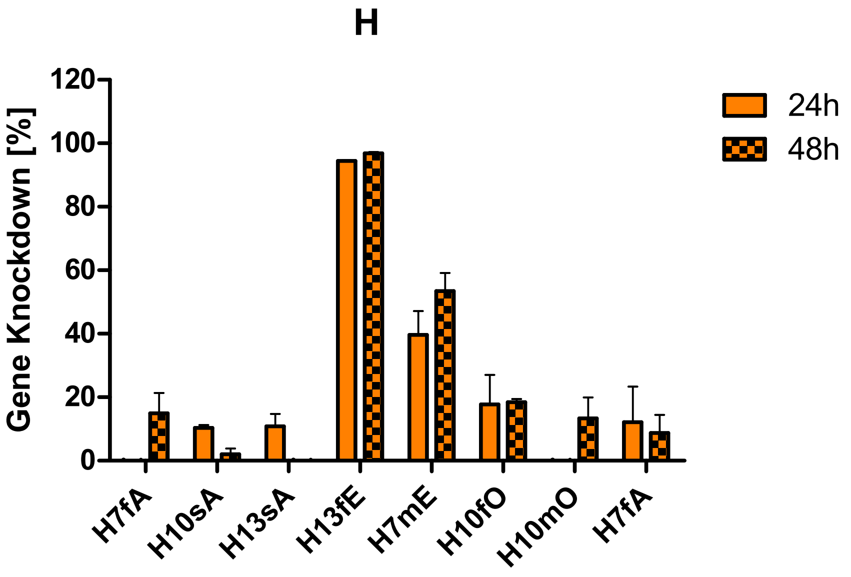

Supplementary Figure 8.: Gene knockdown of different formulations of the hydrophilic polymer (H) after 24 h and 48 h at 50 nM in H1299 Luc cells. Error bars depicting standard error of technical triplicates (n=3).

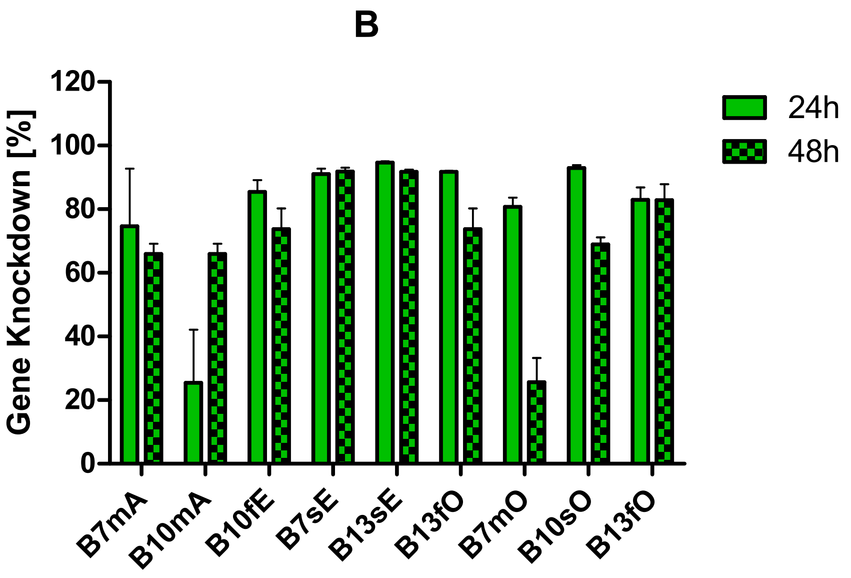

Supplementary Figure 9.: Gene Knockdown of different formulations of the balanced polymer (B) after 24 h and 48 h at 50 nM in H1299 Luc cells. Error bars depicting standard error of technical triplicates (n=3).

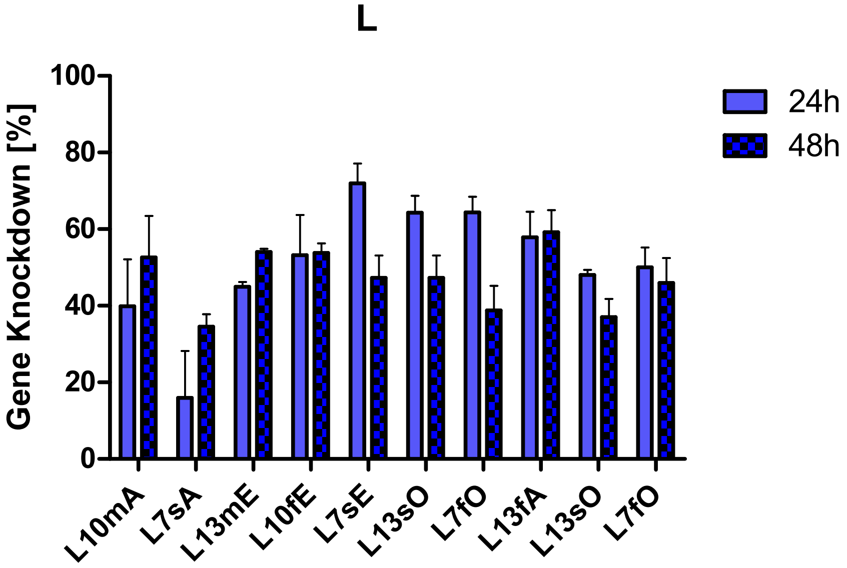

Supplementary Figure 10.: Gene Knockdown of different formulations of the lipophilic polymer (L) after 24 h and 48 h at 50 nM in H1299 Luc cells. Error bars depicting standard error of technical triplicates (n=3).

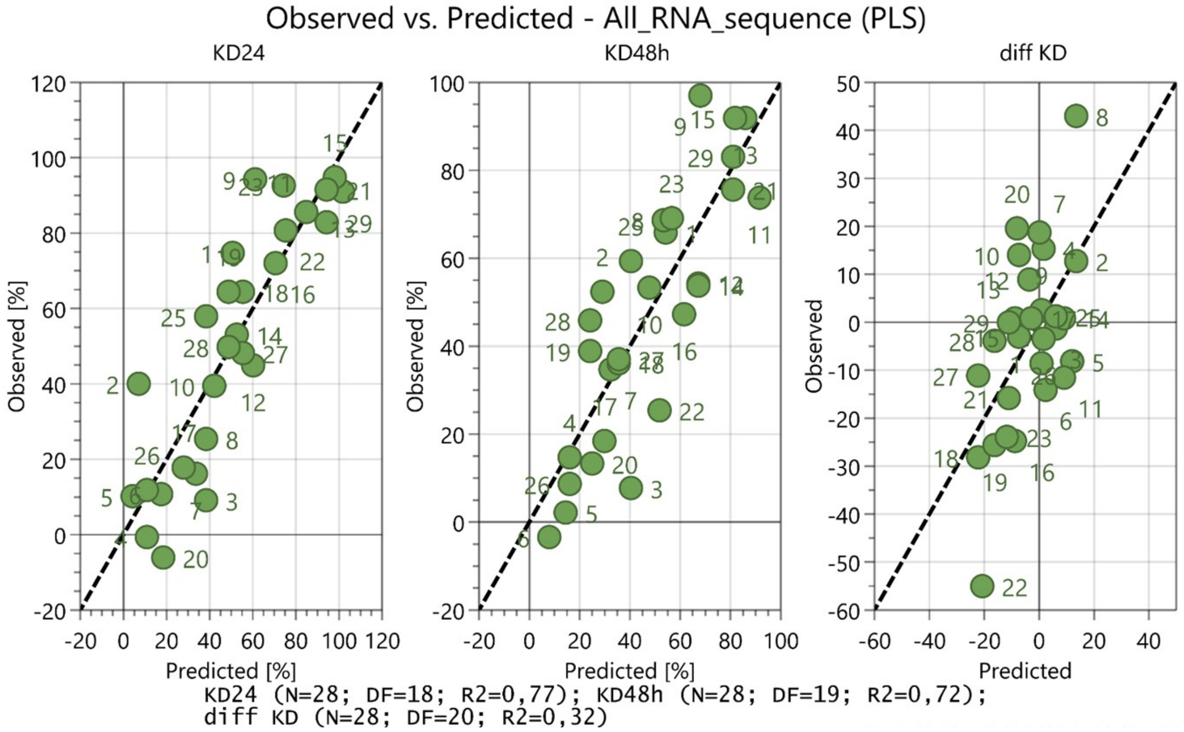

Supplementary Figure 11.: Observed vs Predicted Plots for gene knockdown models after 24 h, 48 h and difference between them, presenting the percentual KD difference.

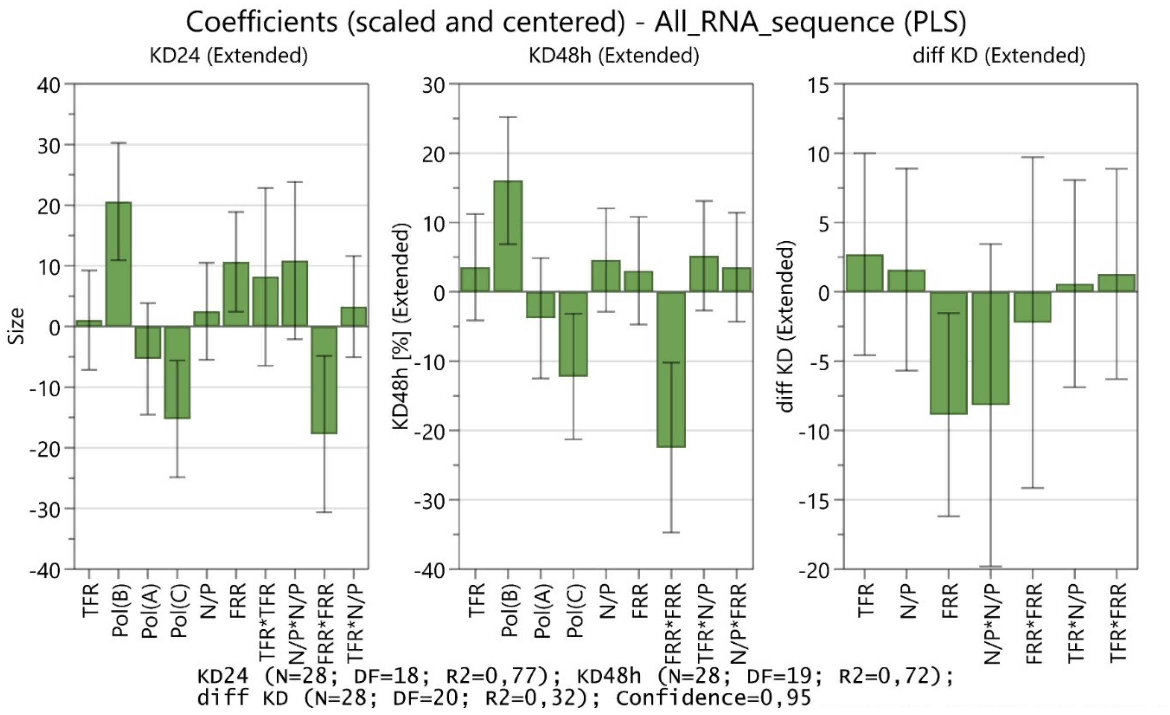

Supplementary Figure 12.: Coefficient plots for gene knockdown models after 24 h, 48 h and the difference between them, presenting the percentual KD difference.

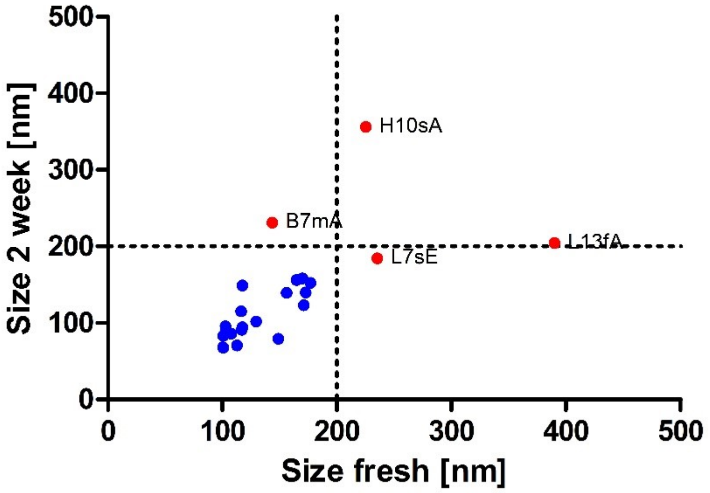

Supplementary Figure 13.: Size correlation of nanoparticles prepared with siLUC freshly prepared or after 2 weeks of storage at 4°C.

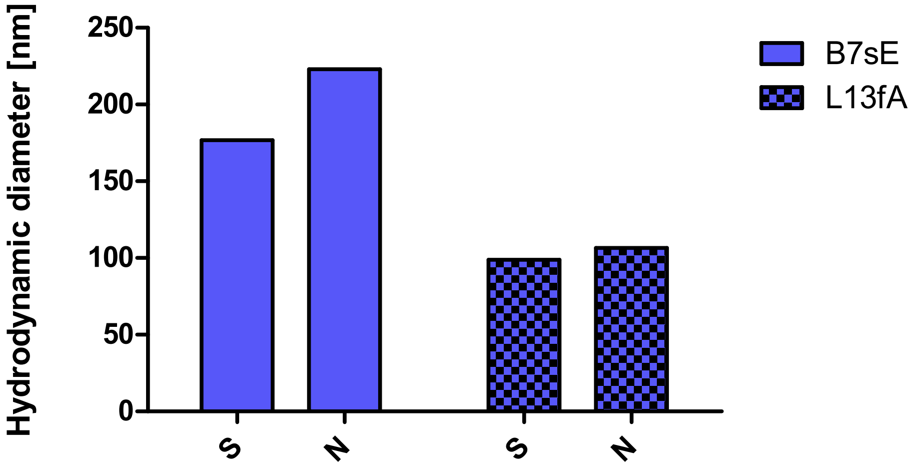

Supplementary Figure 14.: Size of lead formulation (B7sE) and comparison formulation (L13fA) freshly prepared with the Sunscreen Microfluidic device (S) or the Knauer NanoScaler jet impingement mixer (N).

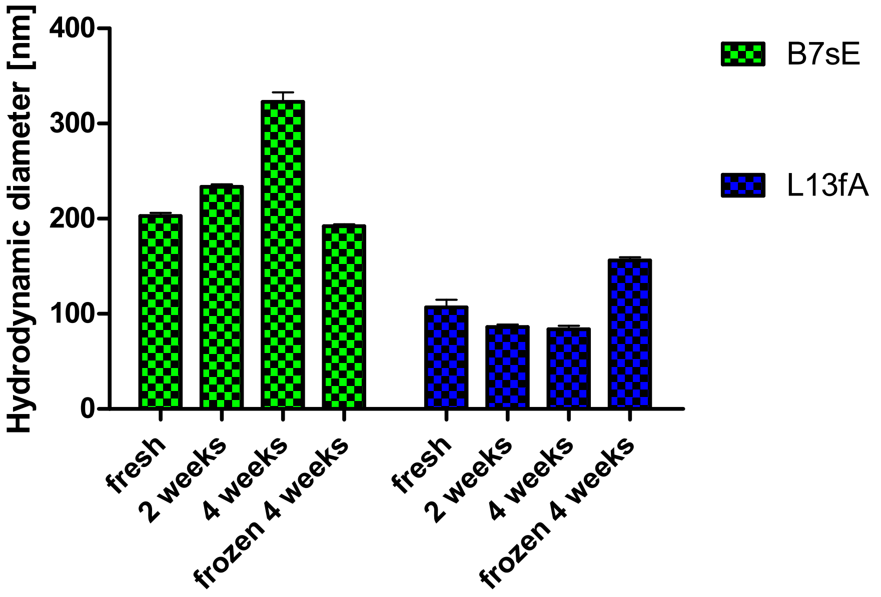

Supplementary Figure 15.: Size of lead formulation (B7sE) and comparison formulation (L13fA) freshly prepared and after storage at 4°C. (Error bars indicate standard deviation, n=3)

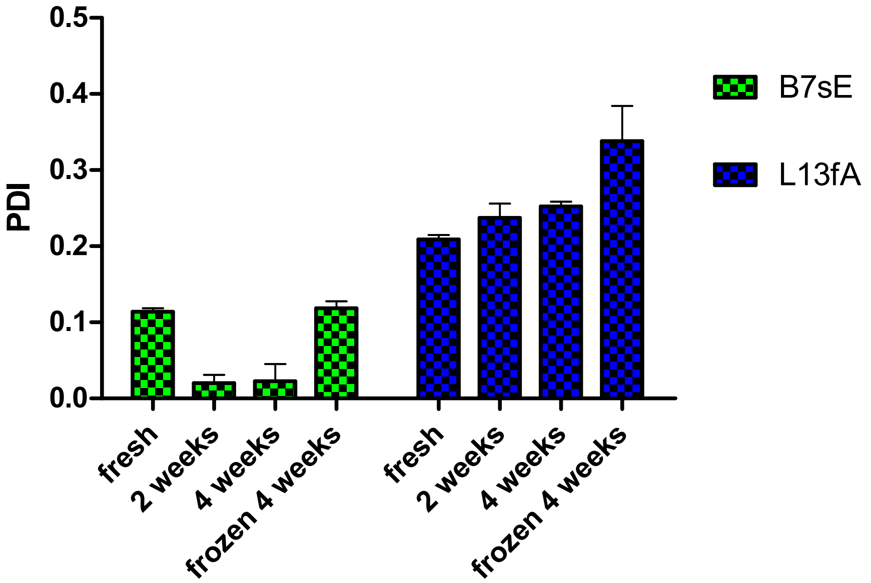

Supplementary Figure 16.: PDI of lead formulation (B7sE) and comparison formulation (L13fA) freshly prepared and after storage at 4°C. (Error bars indicate standard deviation, n=3)

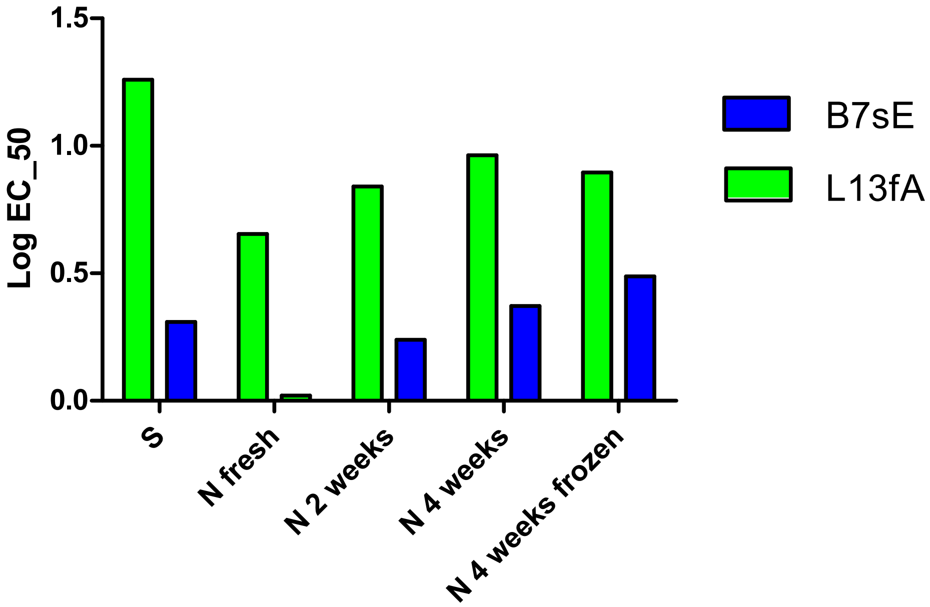

Supplementary Figure 17.: Log EC50 of lead formulation (B7sE) and comparison formulation (L13fA) freshly prepared and after storage at 4°C.

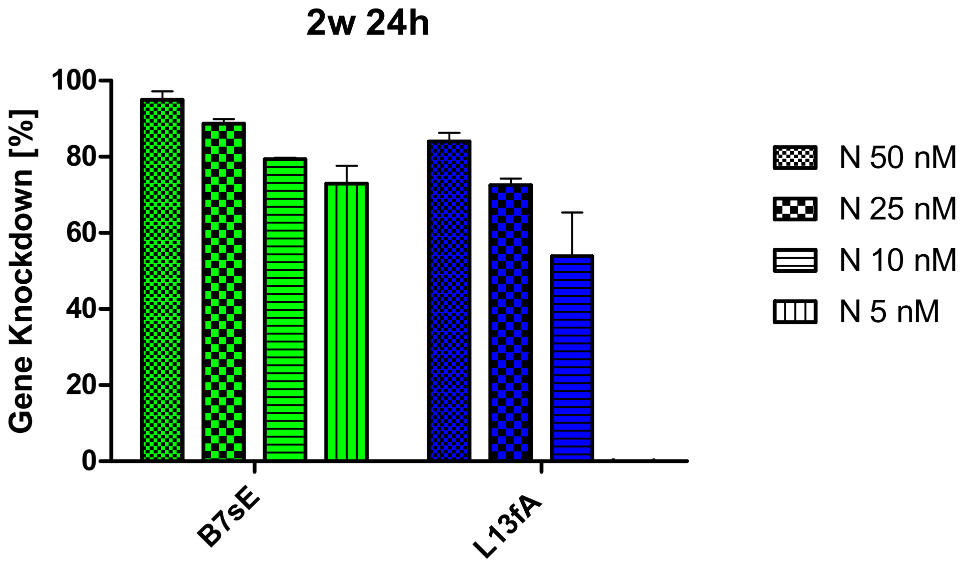

Supplementary Figure 18.: Gene knockdown 24 h after transfection with lead formulation (B7sE) and comparison formulation (L13fA) at different concentrations after 2 weeks of storage at 4 °C (Error bars indicate standard deviation, n=3).

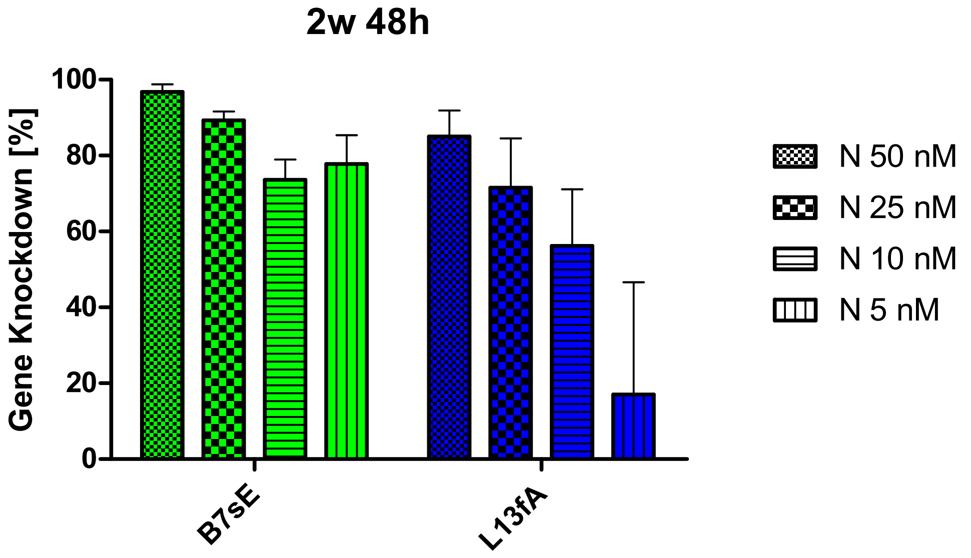

Supplementary Figure 19.: Gene knockdown 48 h after transfection with lead formulation (B7sE) and comparison formulation (L13fA) at different concentrations after 2 weeks of storage at 4 °C (Error bars indicate standard deviation, n=3).

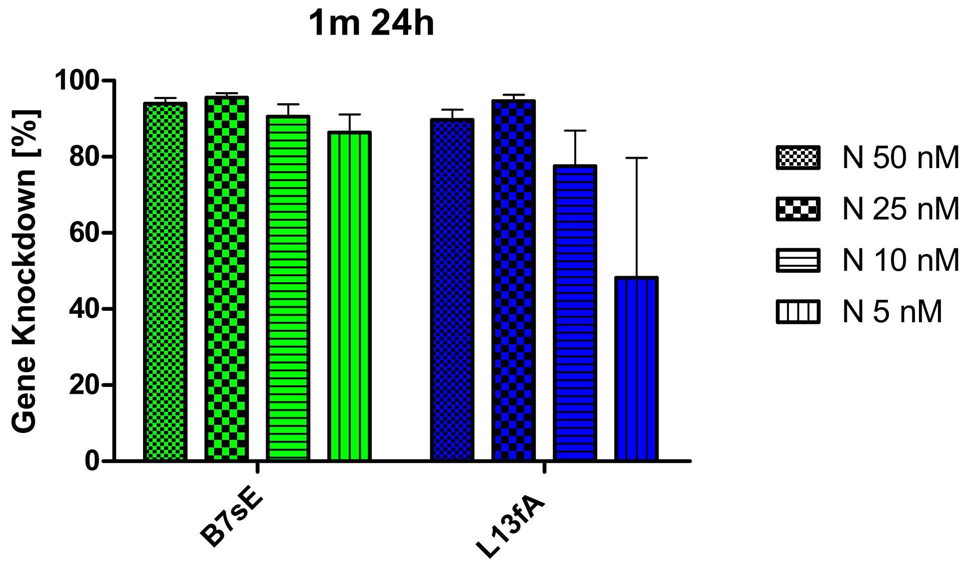

Supplementary Figure 20.: Gene knockdown 24 h after transfection with lead formulation (B7sE) and comparison formulation (L13fA) at different concentrations after 1 month of storage at 4 °C (Error bars indicate standard deviation, n=3)

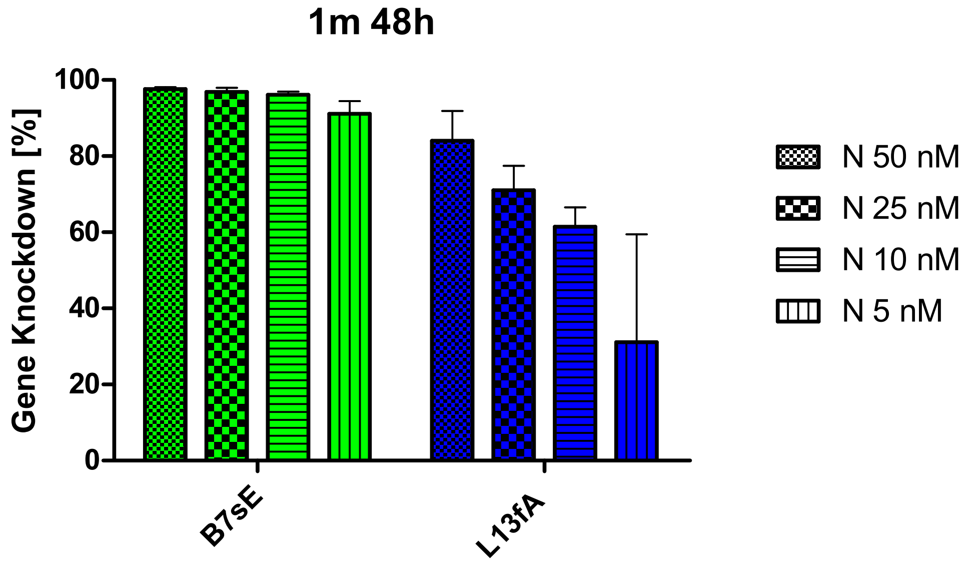

Supplementary Figure 21.: Gene knockdown 48 h after transfection with lead formulation (B7sE) and comparison formulation (L13fA) at different concentrations after 1 month of storage at 4 °C (Error bars indicate standard deviation, n=3)

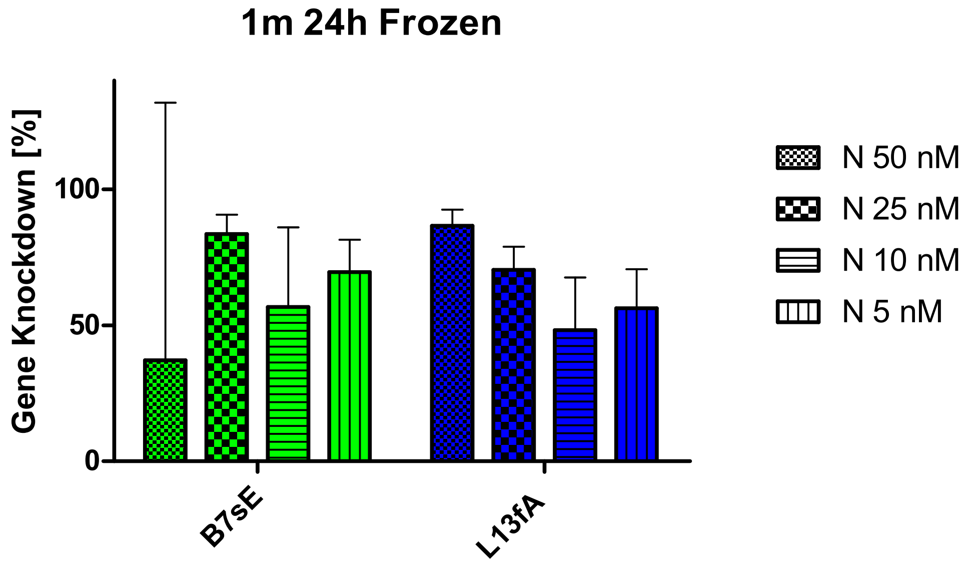

Supplementary Figure 22.: Gene knockdown 24 h after transfection with lead formulation (B7sE) and comparison formulation (L13fA) at different concentrations after 1 month of storage at -20 °C (Error bars indicate standard deviation, n=3)

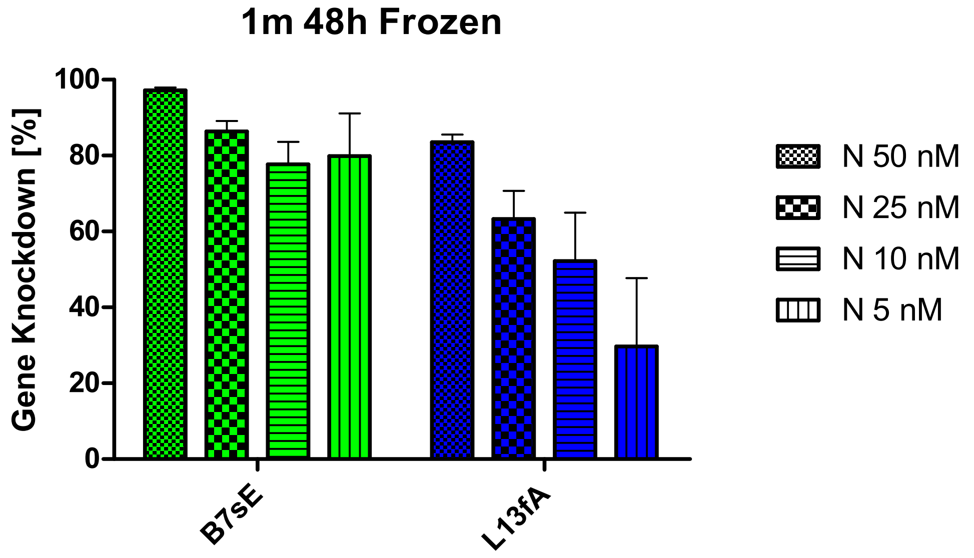

Supplementary Figure 23.: Gene knockdown 48 h after transfection with lead formulation (B7sE) and comparison formulation (L13fA) at different concentrations after 1 month of storage at -20 °C (Error bars indicate standard deviation, n=3)
